# GABA-Induced Exosomes Improve Memory Impairment in Aged Mice

**DOI:** 10.3390/ijms27062519

**Published:** 2026-03-10

**Authors:** Yukina Akama, Shunsuke Maeda, Miyako Udono, Utano Nakamura, Yusuke Yamashita, Youngil Kim, Bungo Shirouchi, Kiichiro Teruya, Yoshinori Katakura

**Affiliations:** 1 Graduate School of Bioresources and Bioenvironmental Sciences, Kyushu University, Fukuoka 819-0395, Japan; akama.yurina.414@s.kyushu-u.ac.jp (Y.A.); 3sl21055g@s.kyushu-u.ac.jp (S.M.); 2 Faculty of Agriculture, Kyushu University, Fukuoka 819-0395, Japan; mudono@grt.kyushu-u.ac.jp (M.U.); kteruya@grt.kyushu-u.ac.jp (K.T.); 3 Pharma Foods International Co., Ltd., Kyoto 615-8245, Japan; u-nakamura@pharmafoods.co.jp (U.N.); y-yamashita@pharmafoods.co.jp (Y.Y.); youngil-kim@pharmafoods.co.jp (Y.K.); 4 Faculty of Nursing and Nutrition, University of Nagasaki, Nagasaki 851-2195, Japan; bshirouchi@sun.ac.jp

**Keywords:** gut–brain interaction, exosome, GABA, memory, aged mice

## Abstract

Gamma-aminobutyric acid (GABA) has been implicated in gut–brain interactions and neuronal activation. We hypothesized that GABA could ameliorate memory decline. We investigated whether oral GABA administration ameliorated age-related cognitive decline in aged mice (C57BL/6J, male) and explored the role of circulating exosomes in mediating these effects. Aged mice that drank water containing 0.5% GABA exhibited significantly improved discrimination index scores compared with that of controls, indicating enhanced memory function. Their plasma-derived exosomes induced neurite outgrowth and mitochondrial activation and restored neuronal activity in SH-SY5Y cells. GABA enhanced the exosomal expression of several miRNAs linked to neuronal activation, longevity, and anti-senescence pathways. Plasma-derived exosomes also restored object recognition memory, reduced hippocampal neuroinflammation, and decreased senescent cell markers (p21 and γH2AX) in aged mice. Additionally, mitochondria- and neurite-related genes were upregulated, and pathways associated with oxidative phosphorylation and Alzheimer’s disease were enriched. Collectively, long-term GABA administration was found to improve cognitive function of aged mice through the secretion of functional exosomes.

## 1. Introduction

Aging is accompanied by a progressive decline in cognitive function, particularly in memory, which is strongly associated with hippocampal neuroinflammation, mitochondrial dysfunction, and cellular senescence [1,2]. Dietary factors can modulate brain health and delay age-related impairment [3]. Among these, gamma-aminobutyric acid (GABA), a non-protein amino acid widely present in fermented foods and plants, is as a bioactive compound with neuroprotective and anti-aging properties [4]. Dietary GABA exerts physiological effects in humans, such as relaxation and immune enhancement [5], and ameliorates cognitive deficits in animal models of Alzheimer’s disease [6]. Activation of the CREB–BDNF signaling axis, together with enhanced mitochondrial function, promotes neurite outgrowth and synaptic plasticity by supporting activity-dependent transcription and energetic demands, thereby strengthening molecular memory mechanisms that underlie memory consolidation and cognitive improvement [7,8].

Our in vitro work further demonstrated that GABA stimulates intestinal epithelial cells to secrete exosomes, which can activate neuronal cells, suggesting a novel mechanism for gut–brain interaction [9,10]. Exosomes are nanosized extracellular vesicles containing proteins, lipids, and microRNAs (miRNAs) that are recognized as important mediators of intercellular communication [11,12]. Dietary components can regulate exosomal composition and function, thereby influencing host physiology and brain health [13]. Importantly, exosomes have been implicated in neuronal plasticity, mitochondrial activity, and neuroinflammation, with increasing evidence supporting their roles in age-related neurodegeneration [14,15,16,17]. However, the functional roles of exosomes derived from aged organisms, particularly during dietary interventions, remain poorly understood.

We hypothesized that oral GABA administration could ameliorate memory decline in aged mice by modulating circulating exosomes. To test this hypothesis, we examined the effects of exosomes derived from aged mice administered GABA on object recognition memory, hippocampal inflammation, senescence, and gene expression in aged mice. This study provides novel insights into the exosome-mediated function of GABA in the regulation of brain function and its underlying molecular mechanisms.

## 2. Results

### 2.1. Effects of GABA on Age-Related Memory Impairment in Aged Mice

**Figure 1:**
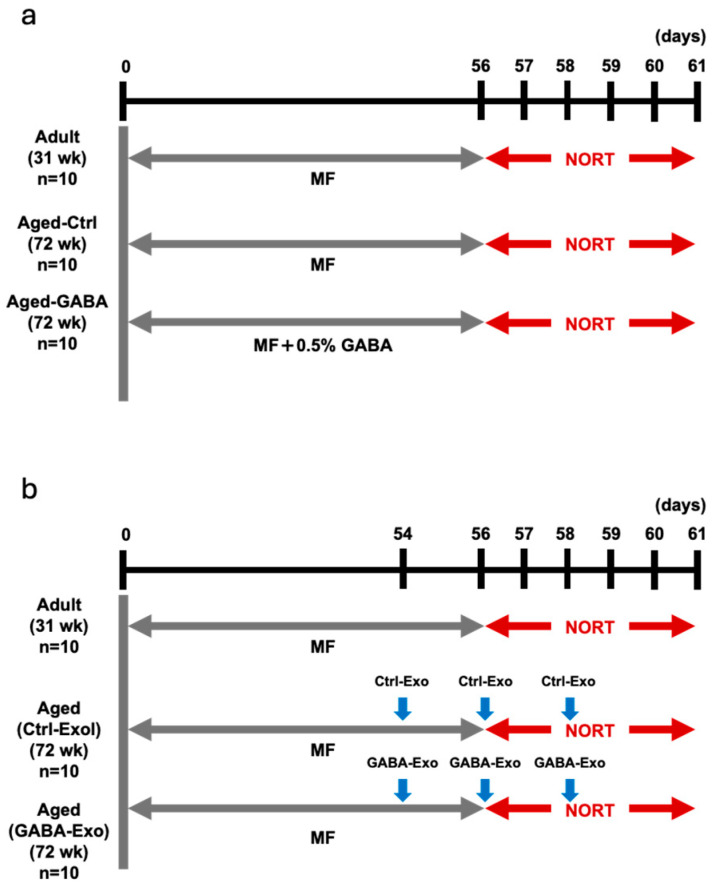
Schematic diagram of the experimental protocol. (**a**) Experimental protocol for NORT following oral administration of the sample. (**b**) Experimental protocol for NORT following exosome administration via the tail vein. NORT: Novel object recognition test.

**Figure 2:**
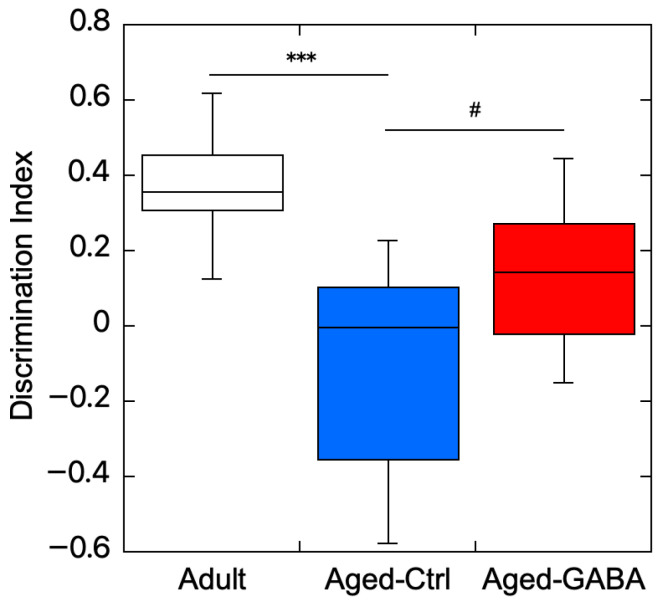
Effects of GABA on age-related memory impairment in aged mice. Exploration preference to novel or familiar objects among Adult, Aged-Ctrl, and Aged-GABA groups were compared. The discrimination index (DI) is shown (*** *p* < 0.001 vs. Adult mice; # *p* < 0.05 vs. Aged-Ctrl mice; data are represented as means ± SEM, *n* = 10). Group differences were evaluated using the Kruskal–Wallis test followed by Dunn’s multiple comparisons test with Holm correction. All tests were two-tailed. GABA: Gamma-aminobutyric acid.

Our previous in vitro studies have shown that GABA activates gut–brain interaction by stimulating the secretion of exosomes, which can activate neuronal cells from intestinal cells [9,10]. We attempted to clarify whether GABA ingestion ameliorates memory impairment in aged mice using in vivo studies and performed the novel object recognition test (NORT) with aged mice. The detailed protocol is described in Figure 1a [18]. In the control (Aged-Ctrl) group, which drank plain water for 56 days, the time spent interacting with familiar objects did not differ significantly from the time spent interacting with unfamiliar objects, whereas the Aged-GABA group, which drank water containing 0.5% GABA for 56 days, showed greater interest in novel objects than in familiar objects. The object discrimination index (DI) score was significantly higher in the Aged-GABA group than that in the Aged-Ctrl group (Figure 2). These results indicate that the long-term ingestion of GABA improves object recognition memory in aged mice.

### 2.2. Functional Evaluation of Plasma Exosomes Derived from GABA-Administered Aged Mice

**Figure 3:**
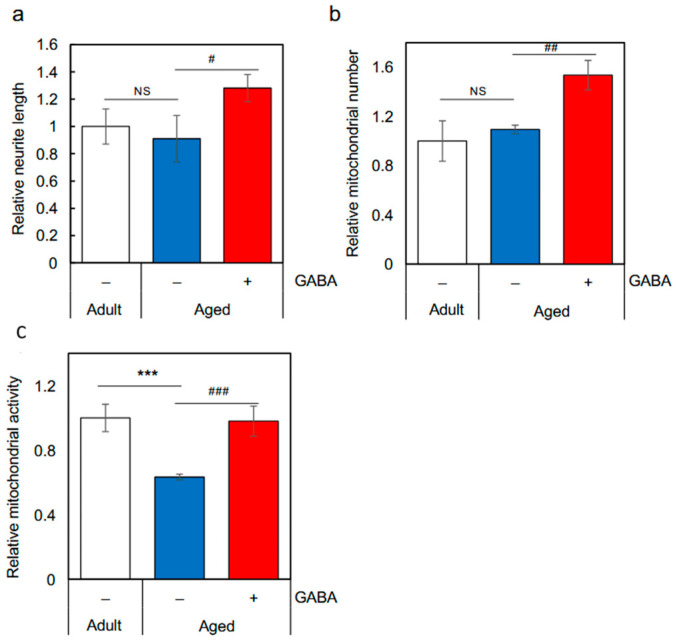
Functional evaluation of plasma exosomes derived from the GABA-fed aged mice. After seeding onto a µClear fluorescence black plate, SH-SY5Y cells were treated with 900 ng/mL exosomes for 24 h and used in the subsequent experiments. (**a**) Effects of plasma exosomes derived from the GABA-fed aged mice on the neurite growth of SH-SY5Y cells. (**b**,**c**) Effects of plasma exosomes derived from the GABA-fed aged mice on the number and activity of mitochondria (*** *p* < 0.001 vs. Adult mice; # *p* < 0.05; ## *p* < 0.01; ### *p* < 0.001 vs. Aged-Ctrl mice; data are represented as means ± SEM, *n* = 10). Experiments were repeated three times, and representative data are shown. GABA: Gamma-aminobutyric acid. NS: not significant.

GABA administration in aged mice improved memory function, and based on our previous findings that plasma-derived exosomes from GABA-administered adult mice can activate human neuronal SH-SY5Y cells we tested whether exosomes derived from the plasma of GABA-fed aged mice could activate SH-SY5Y cells. Several instances have been reported where mouse-derived exosomes exhibit functional effects on human cells, and cases have also been documented where mouse-derived exosomes are taken up by human cells [9,19]. Physicochemical properties of the exosomes isolated from plasma were evaluated by NanoSight (Figure S1). Figure 3 shows the activities of exosomes derived from adult mice and aged mice that drank either plain water or GABA-containing water (Figure 3a–c). Exosomes derived from the plasma of GABA-fed aged mice significantly induced neurite growth and caused an increase in the number and activation of mitochondria in SH-SY5Y cells compared with those derived from the plasma of control aged mice. The activities of the exosomes derived from the plasma of GABA-fed aged mice were comparable to or even stronger than those derived from the plasma of adult mice. Previous reports indicate that phosphodiesterase inhibitors enhance mitochondrial activity in SH-SY5Y cells, activate mitochondria in the mouse hippocampus, and improve memory function [20]. Furthermore, Wogonin simultaneously induces neurite outgrowth in SH-SY5Y cells and improves memory function in mice [21], and similar functionality is thought to be demonstrated for GABA. A comparison of the effects of plasma exosomes derived from control aged mice versus adult mice on SH-SY5Y cells revealed no significant differences in neurite outgrowth or mitochondrial number. However, mitochondrial activity was reduced in control aged mice. The cause of this difference in activity must be clarified in the future.

### 2.3. Pathways Regulated by miRNAs Derived from Plasma Exosomes of Aged-GABA Mice

**Figure 4:**
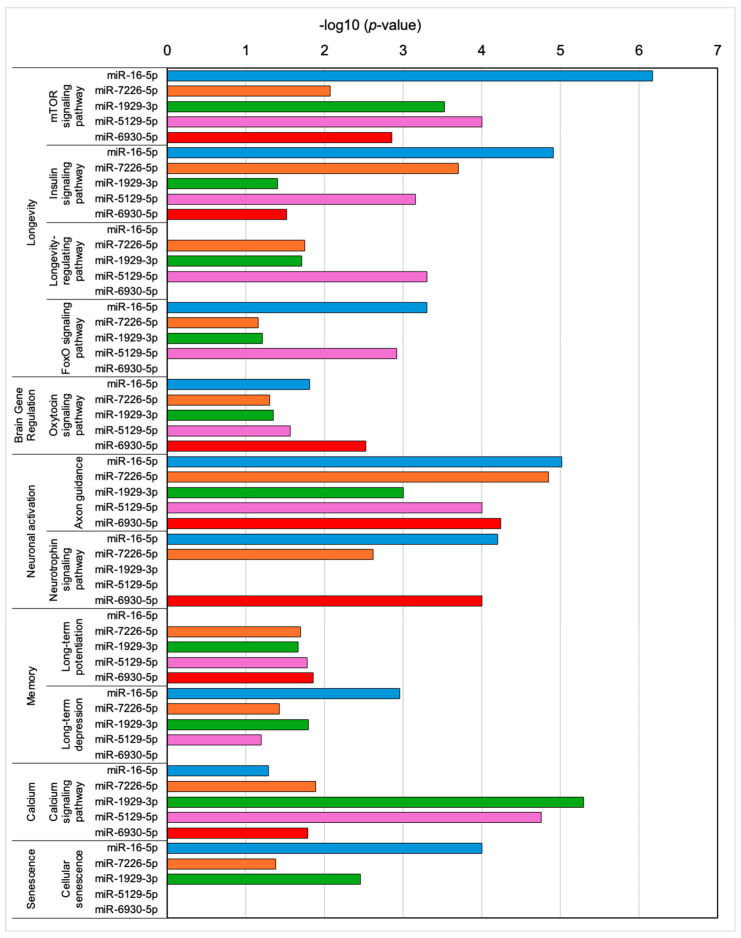
Pathways regulated by miRNAs derived from plasma exosomes of GABA-fed mice. The expression profiles of miRNAs in plasma exosomes after GABA ingestion were analyzed using a microarray and the expression of five miRNAs (miR-16-5p, miR-7226-5p, miR-1929-3p, miR-5129-5p, and miR-6930-5p) was enhanced in plasma exosomes of GABA-fed mice. By using TargeScan (Release 8.0) and DAVID 2021, the pathways regulated by the target genes were estimated. GABA: Gamma-aminobutyric acid.

To investigate the possibility of neuronal activation by exosomes, we analyzed the expression profiles of miRNAs in plasma exosomes after GABA ingestion and identified their target genes to estimate their functions. Compared to the Aged-Ctrl group, we selected the five most highly expressed miRNAs (miR-16-5p, miR-7226-5p, miR-1929-3p, miR-5129-5p, and miR-6930-5p) in plasma-derived exosomes from Aged-GABA mice and performed analysis (Figure 4). Compared to Aged-Ctrl mice, the relative global normalization values for miR 16-5p, miR-7226-5p, miR-1929-3p, miR-5129-5p, and miR-6930-5p were 2.06, 2.25, 33.71, 2.29, and 2.12, respectively. The expression these five miRNAs in exosomes was verified by RT-qPCR and shown in Figure S2a. Furthermore, based on the results in Figure 4, it was inferred that miR-16-5p and miR-6930-5p are involved in regulating brain and neural functions. Therefore, using mimics, we evaluated their contribution to neuronal function by using neurite outgrowth as an indicator. The results clearly demonstrated that these miRNAs are indeed involved in regulating neuronal function (Figure S2b). Next, we used TargetScan (Release 8.0) to identify the target genes of each miRNA and DAVID to estimate the pathways regulated by the target genes. Various pathways related to longevity, brain gene regulation, neuronal activation, memory, calcium, and senescence were regulated by enhanced miRNAs (Figure 4). GABA ingestion may cause exosomes that regulate aging, longevity, and brain function to flow into the bloodstream, resulting in the regulation of brain function.

### 2.4. Exosomes Derived from Aged-GABA Mice Recovered Object Recognition Memory and Hippocampal Status in Aged Mice

**Figure 5:**
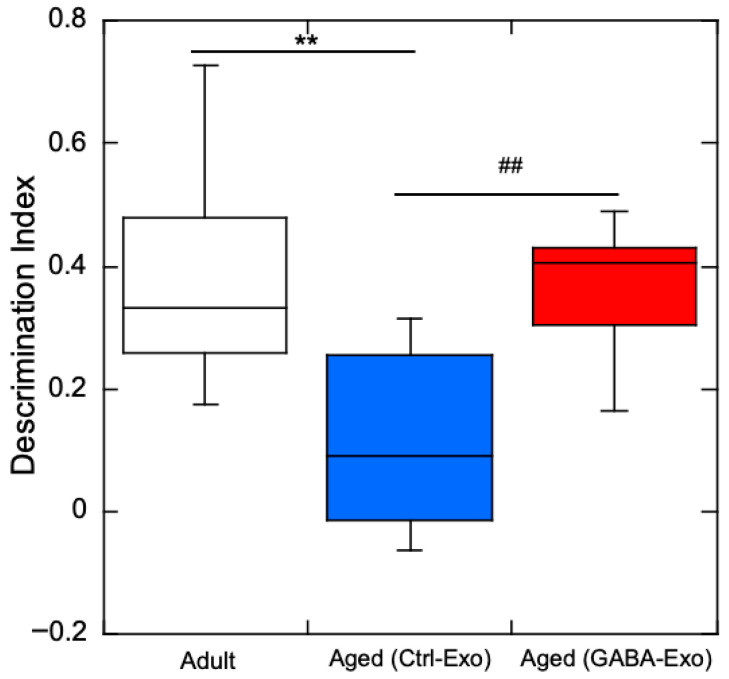
Exosomes derived from GABA-fed aged mice recovered object recognition memory. Exploration preference to novel or familiar objects among Adult, Aged (Ctrl-Exo), and Aged (GABA-Exo) groups were compared. The discrimination index (DI) is shown (** *p* < 0.01 vs. Adult mice; ## *p* < 0.01 vs. Aged (Ctrl-Exo) mice; data are represented as means ± SEM, *n* = 10). GABA: Gamma-aminobutyric acid.

GABA restores object recognition memory in aged mice, and plasma exosomes of GABA-fed mice can activate neuronal cells. We tested whether plasma exosomes from Aged-GABA mice could restore object recognition memory in aged mice (Figure 1b). Adult mice were included as a physiological age reference group to provide baseline cognitive and molecular profiles. In addition to the NORT, anxiety-like behavior was evaluated using the open field test, in which reduced center exploration reflects increased anxiety. No significant difference was observed in the time spent in the central area or the number of entries into the central area, and there was no significant change in anxiety-like behavior in these mice. The DI value was significantly decreased for the Aged (Ctrl-Exo) group, in which exosomes derived from the plasma of Aged-Ctrl mice were injected into the tail vein, compared with that of adult mice, indicating that object recognition memory was impaired in aged mice, even after the plasma exosomes of aged mice administered sterile water were injected (Figure 5). However, compared with the Aged (Ctrl-Exo) group, the Aged (GABA-Exo) group, in which plasma exosomes derived from Aged-GABA mice were administered via the tail vein, showed a significant increase in DI value, indicating that object recognition memory was recovered by the injection of exosomes from Aged-GABA mice.

**Figure 6:**
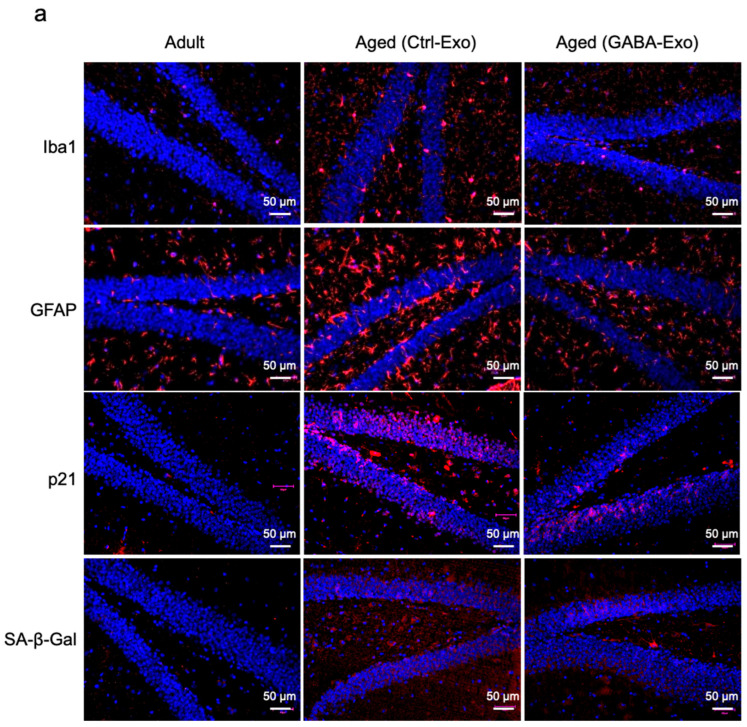

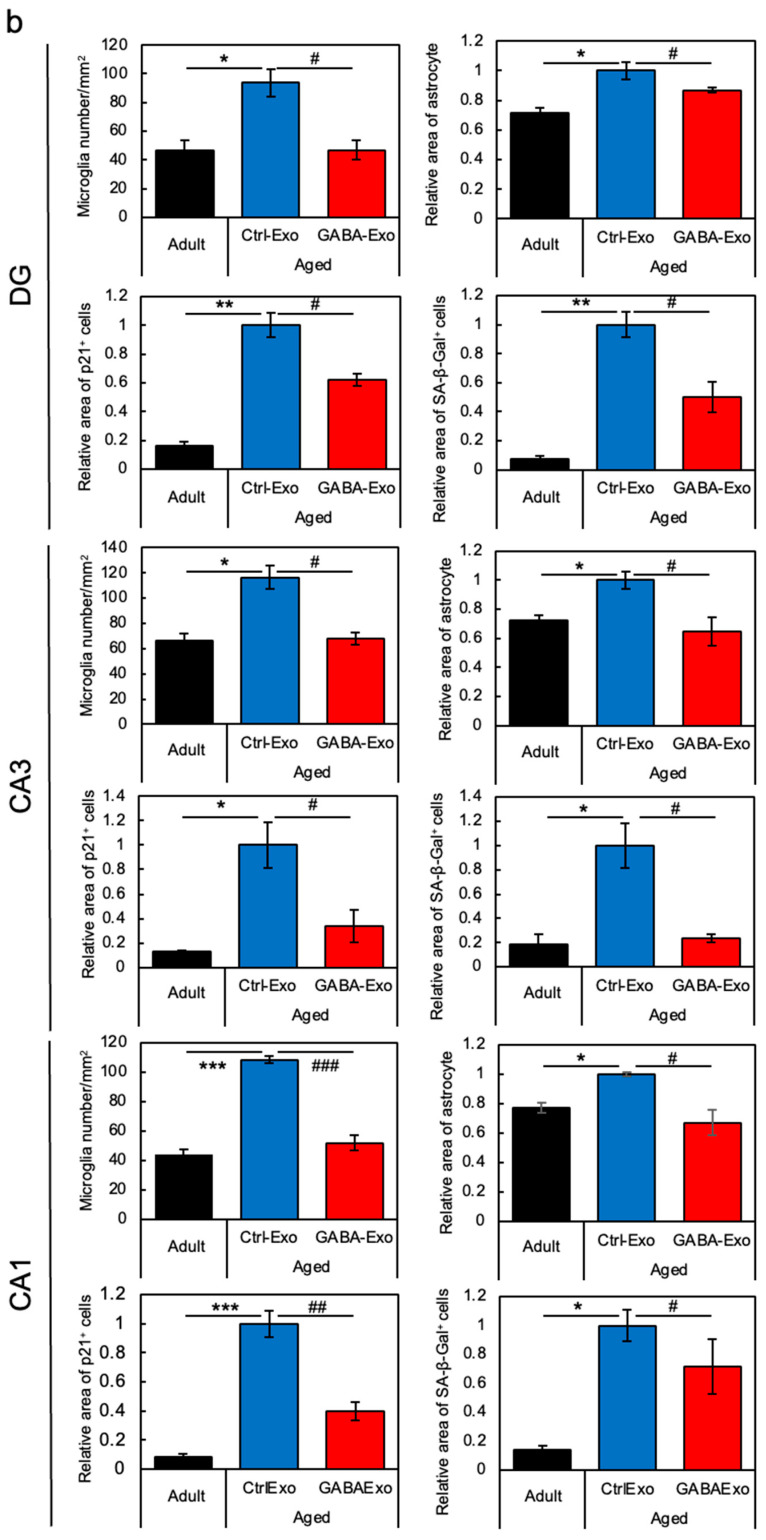
Effects of exosomes derived from GABA-fed mice on hippocampus in aged mice. (**a**) Brain sections (dentate gyrus, DG) were incubated with primary antibody for anti-Iba1, anti-GFAP, anti-p21 and anti-β-galactosidase, and stained with Alexa Fluor 555. After staining with Vestashield mounting medium, the tissue samples were observed under a fluorescence microscope. A 50 μm scale bar is shown in the lower right corner of each photograph. (**b**) Number of Iba1^+^ cells per area and relative area of GFAP^+^ cells, p21^+^ cells and SA-β-Gal^+^ cells in DG, CA3 and CA1 are shown as bar graphs (* *p* < 0.05; ** *p* < 0.01; *** *p* < 0.001 vs. Adult mice; # *p* < 0.05; ## *p* < 0.01; ### *p <* 0.001 vs. Aged (Ctrl-Exo) mice; data are represented as means ± SEM, *n* = 10). GABA: Gamma-aminobutyric acid.

We tested the effects of exosomes derived from Aged-GABA mice on hippocampal inflammation and gene expression. Glial cells, such as microglia and astrocytes, are the source of various mediators that significantly contribute to neuroinflammation, oxidative brain damage, and neuronal apoptosis [22,23,24]. Therefore, we used Iba-1 and GFAP as markers to verify the activation status of the microglia and astrocytes [18]. All brain sections were collected according to anatomical landmarks, and quantitative analyses were performed within defined hippocampal subregions to ensure consistency across groups. Activated microglia and astrocytes increased in the dentate gyrus (Figure 6a,b), CA3 (Figure 6b and Figure S3), and CA1 (Figure 6b and Figure S4) of the hippocampus of Aged (Ctrl-Exo) mice compared with those in adult mice. In particular, an increase in the number of Iba-1-positive cells with amoeboid morphology revealed enhanced neuroinflammation. Conversely, there was a significant decrease in the numbers of activated microglia (Figure 6a,b and Figures S3 and S4) and astrocytes (Figure 6a,b and Figures S3 and S4) in Aged (GABA-Exo) mice. Plasma exosomes derived from GABA-fed aged mice reduced the number of Iba-1-positive microglia and GFAP-positive astrocytes in the hippocampus of aged mice, and further decreased neuroinflammation.

Previous reports have shown that the number of cells positive for cellular senescence markers, including p21 and senescence-associated β-galactosidase (SA-β-Gal), in the hippocampus increases with age [25,26]. We examined the effect of exosomes derived from Aged-GABA mice on cellular senescence in the hippocampus. The number of p21- and SA-β-Gal-positive cells was significantly increased in Aged (Ctrl-Exo) mice compared with adult mice (Figure 6a,b and Figures S3 and S4). However, the number of p21- and SA-β-Gal-positive cells was significantly reduced in Aged (GABA-Exo) mice compared with Aged (Ctrl-Exo) mice. These results indicated that the number of senescent cells in the hippocampus of aged mice was reduced by the administration of exosomes derived from Aged-GABA mice.

**Figure 7:**
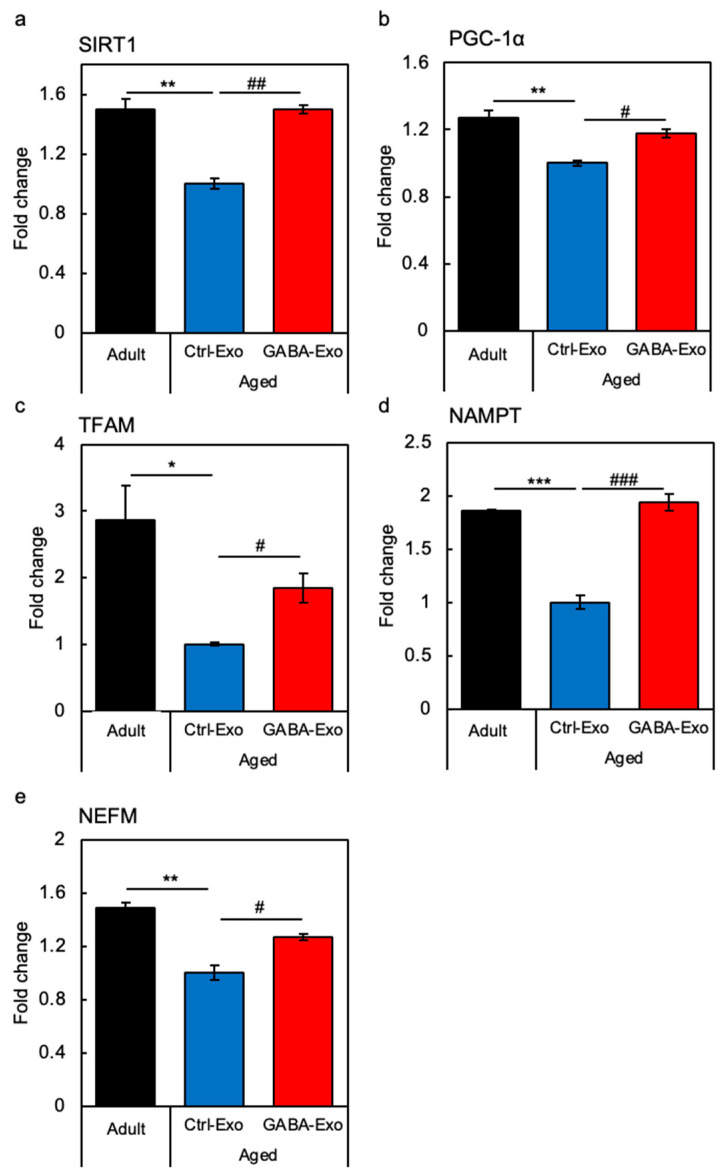
Effects of exosomes derived from GABA-fed mice on gene expression in the hippocampus of aged mice. The expression of genes (SIRT1 (**a**), PGC-1α (**b**), mitochondrial transcription factor A [TFAM] (**c**), nicotinamide phosphoribosyltransferase [NAMPT] (**d**), neurofilament medium chain [NEFM] (**e**)) in the hippocampus of adult mice (Adult), aged mice injected with control exosomes (Aged Ctrl-Exo), and aged mice injected with exosomes derived from GABA-fed aged mice (Aged GABA-Exo) were analyzed using RT-qPCR (* *p* < 0.05; ** *p* < 0.01; *** *p* < 0.001 vs. Adult mice; # *p* < 0.05; ## *p* < 0.01; ### *p* < 0.001 vs. Aged (Ctrl-Exo) mice; data are represented as means ± SEM, *n* = 3). GABA: Gamma-aminobutyric acid.

Next, we tested the effect of tail vein injection of exosomes from Aged-GABA mice on the expression of neurite growth- and mitochondria-related genes in the hippocampus. The expression of all genes was significantly decreased in the Aged (Ctrl-Exo) mice, but the expression of these genes was significantly restored by the injection of exosomes from Aged-GABA mice, to levels similar to those in adult mice (Figure 7a–e). We intend to elucidate the relationship between these genes and the fluctuating miRNAs in the future.

### 2.5. Comprehensive Analysis of Gene Expression in the Hippocampus

We analyzed the changes in gene expression in the hippocampus of aged mice injected with exosomes from aged mice administered GABA or sterile water. RNA-seq analysis revealed a significant change in gene expression in the hippocampus of Aged (GABA-Exo) mice compared with that in Aged (Ctrl-Exo) mice, with a decrease in the expression of 505 genes and an increase in the expression of 1198 genes. Next, the pathways involving genes whose expression varied were established using iDEP2.0. These differentially expressed genes were significantly involved in the oxidative phosphorylation and Alzheimer’s disease pathways (Figures S5 and S6). These results may correspond to the enhanced expression of mitochondria-related genes and improved memory function in the hippocampus of Aged (GABA-Exo) mice. Furthermore, transcriptome-wide comparison between Aged (Ctrl-Exo) and Aged (GABA-Exo) hippocampi identified extensive differential gene expression. Pathway enrichment analysis revealed significant modulation of multiple biological processes, including glycogen and glucan biosynthetic and metabolic pathways, regulation of polysaccharide metabolism, calcineurin–NFAT signaling, and inositol-phosphate-mediated signaling (Figure S7). These pathways are known to be critically involved in neuronal energy metabolism, activity-dependent signaling, and synaptic function, providing broader mechanistic context for the observed cognitive improvement.

**Figure 8:**
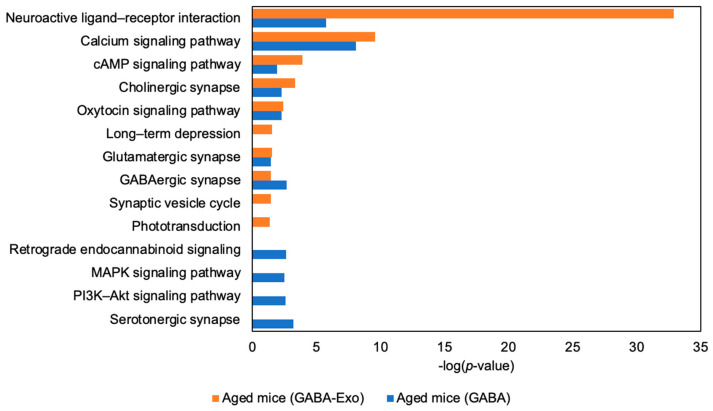
Gene expression in the hippocampi of GABA-fed aged mice (Aged mice (GABA)) and Aged mice (GABA-Exo). Microarray analysis identified genes exhibiting expression changes in the hippocampus of each mouse. The pathways regulated by these differentially expressed genes were analyzed using DAVID, and pathways considered to be involved in neural and brain function control were shown. Blue bars indicate Aged mice (GABA), while orange bars indicate Aged mice (GABA-Exo). GABA: Gamma-aminobutyric acid.

We compared gene expression in the hippocampus of Aged-GABA and Aged (GABA-Exo) mice. The expression of genes involved in memory, neuronal function regulation, and calcium pathways were altered in the hippocampus of each mouse (Figure 8). These results suggest that the brain-function-improving effects of GABA are mediated by plasma exosomes. Furthermore, mice administered GABA and mice injected with GABA-treated mouse-derived exosomes via the tail vein exhibited differing degrees of effect intensity. One possible reason for this is that the amounts of exosomes released into the bloodstream differs between oral administration of GABA and direct administration of exosomes via the tail vein.

## 3. Discussion

In the present study, we demonstrated that long-term dietary supplementation with GABA ameliorates age-related memory impairment in mice, and that plasma-derived exosomes play a central role in mediating these effects. Oral GABA administration improved object recognition memory, and exosomes isolated from GABA-fed aged mice restored memory performance when transferred to recipient aged mice. These findings suggest that GABA exerts its neuroprotective actions through direct signaling and exosome-mediated intercellular communication. In the present study, cognitive performance was evaluated using the novel object recognition (NOR) test, which is a well-established paradigm for assessing object recognition memory. However, our behavioral findings primarily reflect changes in recognition memory rather than global cognitive function. Additional behavioral assays such as the Y-maze or Morris water maze would provide complementary assessment of spatial and associative memory. Our experimental design focused on detecting early changes in recognition memory following exosome treatment. Notably, we observed marked suppression of inflammatory markers in the hippocampal CA1 and CA3 regions, which are critically involved in spatial and associative memory, respectively [27,28]. These molecular alterations provide supportive evidence for broader hippocampal functional improvement; however, direct behavioral validation of these domains remains to be established. Future studies incorporating multiple behavioral paradigms will be required to further characterize the effects of exosome treatment across distinct cognitive domains. Furthermore, although the present study focused on early post-intervention outcomes, the long-term durability of the observed effects remains to be determined. Future longitudinal studies will be important to assess whether the cognitive and molecular changes persist over extended periods after the intervention.

Our results are consistent with those of previous reports showing that dietary GABA influences brain function and attenuates cognitive deficits [5,29]. Importantly, we extend these observations by demonstrating that plasma exosomes act as functional mediators carrying a molecular cargo that enhances neuronal activity. Exosomes derived from Aged-GABA mice induced neurite outgrowth and mitochondrial activation in SH-SY5Y cells, which was diminished by exosomes from Aged-Ctrl mice. This aligns with the accumulating evidence that exosomes contribute to neuronal plasticity and mitochondrial regulation during aging [14,30]. Although NanoSight analysis was performed to assess exosome size distribution (Figure S1), additional characterization methods such as TEM-based morphology analysis and proteomic or lipidomic profiling were not conducted in this study. These analyses will be addressed in future study to further elucidate the physicochemical properties of the exosomes. It has been reported that exosomes in the blood cross the blood–brain barrier, reach the brain, and control brain function [30]. Similarly, it is thought that GABA activates the gut–brain axis by mediating the secretion of functional exosomes into the bloodstream. Furthermore, exosomes derived from adult mouse plasma were reported to suppress the age-related decline in mitochondrial energy metabolism [30]. In a future study, we intend to investigate the functional differences and variations in contained miRNAs between exosomes derived from adult mouse plasma and those derived from GABA-fed mouse plasma, and to test the requirement of exosomal miRNAs using depletion or inhibition approaches.

miRNA profiling of plasma exosomes revealed the enrichment of miRNAs associated with neuronal activation, longevity, and anti-senescence pathways. This observation suggests that dietary GABA modifies the exosomal cargo to favor brain-protective signaling. Notably, exosomes from GABA-fed aged mice reduced neuroinflammation, as indicated by decreased Iba-1- and GFAP-positive cells in the hippocampus, and suppressed cellular senescence markers, such as p21 and SA-β-Gal [25,31]. These results provide mechanistic insight into how GABA attenuates hippocampal dysfunction by linking food-derived compounds to the regulation of neuroinflammation and senescence. Furthermore, although no direct evidence exists, among the miRNAs shown in Figure 4, miR-5129-5p has been reported as an intestinal-derived miRNA, and miR-16-5p is an miRNA implicated in associations with enteritis and intestinal epithelial function [32,33]. Therefore, at least some exosomes can be considered to be of intestinal origin. Bioinformatic analyses highlighted several candidate miRNAs that may be involved in cognitive improvement. Future investigations will focus on functional validation of these miRNAs and their target genes, as well as on dissecting the roles of other exosomal components, such as proteins and lipids, in mediating the observed effects.

Furthermore, RNA-seq analysis revealed that exosomes from GABA-fed aged mice altered hippocampal gene expression, particularly in pathways related to oxidative phosphorylation and Alzheimer’s disease. These transcriptional changes were consistent with enhanced mitochondrial activity and improved memory function, suggesting that dietary GABA may mitigate age-related decline by restoring energy metabolism in the brain.

Taken together, our findings highlight a novel gut–brain interaction whereby oral GABA administration modifies plasma exosomes, leading to reduced hippocampal inflammation, suppression of cellular senescence, and improvement of cognitive function in aged mice. Although our study establishes a strong preclinical foundation, several limitations should be considered. First, we did not examine the direct molecular mechanism by which GABA stimulates exosome production in intestinal cells in vivo. Second, the relevance of these findings to human aging and dietary interventions remains to be validated. Future studies should aim to identify specific exosomal miRNA–target interactions, clarify the tissue origins of plasma exosomes, and evaluate their translational potential in human cohorts. In addition, although GABA’s bioavailability is considered low, doses of approximately 100–200 mg per day are expected to be effective for improving brain function in humans. In this study, brain function improvement was observed in mice administered approximately 25 mg per day, suggesting similar efficacy can be anticipated in humans.

In conclusion, this study provides evidence that GABA acts as a functional food compound that can restore brain function during aging through exosome-mediated gut–brain interactions. These results provided new avenues for developing dietary strategies to prevent cognitive decline and promote healthy aging.

## 4. Materials and Methods

### 4.1. Cell Culture and Reagent

Cells from the human neuronal cell line SH-SY5Y (American Type Culture Collection, Manassas, VA, USA) were cultured in Dulbecco’s modified Eagle’s medium (Nissui, Tokyo, Japan) containing 10% heat-treated fetal bovine serum (Capricorn Scientific GmbH, Ebsdorfergrund, Germany) and cultured at 37 °C and 5% CO_2_. GABA was purchased from Abcam (Cambridge, UK).

### 4.2. Animal Experiments

All experiments were conducted in accordance with the Guide for the Care and Use of Laboratory Animals and approved by the Ethics Committee on Animal Experimentation (Kyushu University; approval number: A24-195-1). The criteria for excluding animals from an experiment or analysis are as described in the animal experiment application form, but no adverse events were observed. Adult mice (C57BL/6J, male, 31-week-old, Charles River Laboratories Japan, Osaka, Japan) and aged mice (C57BL/6J, male, 72-week-old, The Foundation of Biomedical Research and Innovation at Kobe through the National BioResource Project of MEXT, Kobe, Japan) were used in the study. Seventy-two-week-old mice represent a biologically and experimentally validated aged stage in which age-related cognitive and molecular impairments are evident but still amenable to intervention [34,35]. Sample size was determined on the basis of an initial investigation conducted to test memory function. Ten mice per group were housed in cages of five mice per cage, maintained on a 12 h light/dark cycle, and allowed ad libitum access to food and water. The mice underwent acclimatization for one week at the designated experimental facility. The assignment of mice to experimental and control groups was random and conducted by a responsible person who was not in charge of the actual experiment. All behavioral procedures were conducted during the light phase of the cycle. The Aged-GABA group was administered water containing 0.5% GABA for 56 days, whereas the Aged-Ctrl group was administered sterile water for 56 days. To minimize potential confounders, treatments and measurements were randomized and carried out in double-blind conditions, and careful consideration was given to the order of measurements, animal placement, and cage positioning. Weight loss exceeding 10% compared to control mice kept without food administration was considered a humane endpoint.

### 4.3. NORT

To clarify the role of ingested GABA and plasma-derived exosomes in object recognition memory formation, the NORT was performed in accordance with a previously described method [36]. For the habituation phase, individual adult male mice were placed in a chamber (40 × 40 × 30 cm) and allowed to explore freely for 10 min over 3 days. In the training phase, mice were placed in the same chamber containing two different objects for 10 min and allowed to explore the objects. After 24 h, during the testing phase, one of the objects was exchanged for a new object, ensuring that the locations of the two objects did not change. The duration of exploratory behavior exhibited by the mice was determined, and exploration preference was calculated. The DI was calculated as follows [18]:

(Time spent exploring the novel object − Time spent exploring the familiar object)/(Time spent exploring the novel object + Time spent exploring the familiar object)

Anxiety-like behavior was assessed using the open field test. Mice were placed in the center of a square arena (40 × 40 cm) and allowed to explore freely for 10 min. The arena was virtually divided into central and peripheral zones. Anxiety-related behavior was evaluated by time spent in the center zone and number of center entries.

### 4.4. Exosome Isolation

After sample administration, blood was collected from the hearts of the anesthetized mice. Exosomes were prepared from plasma samples using the MagCapture Exosome Isolation PS ver 2.0 Kit (FUJIFILM Wako Pure Chemical Corp., Osaka, Japan). Exosome-specific marker proteins were used as indicators for purification via affinity chromatography, ensuring that no non-exosomal components were present [37]. Furthermore, although there is a possibility that peptides may be adsorbed onto the exosomes to a very slight degree, it was considered that free peptides were not present. Exosome quantification was performed using the Micro BCA Protein Assay Kit (Thermo Fisher Scientific, Waltham, MA, USA) as an indicator of protein quantity.

### 4.5. Tail Vein Injection of Exosomes

Plasma-derived exosomes were prepared from aged mice administered GABA for 2 months and injected into the tail vein of aged mice at a dose of 5 µg/100 µL of PBS every other day for three consecutive days. In the control group, plasma-derived exosomes from aged mice administered sterile water were injected into the tail veins of aged mice under the same conditions.

### 4.6. Quantitative Evaluation of Neurite Growth

SH-SY5Y cells were seeded onto a μClear fluorescence black plate (Greiner-Bio One, Tokyo, Japan), fixed with 4% paraformaldehyde for 15 min, and blocked with blocking buffer (1× PBS, 5% goat serum, and 0.3% Triton X-100) for 1 h. The cells were subsequently incubated with Milli-Mark Pan Neuronal Marker (Merck Millipore, Billerica, MA, USA) at 25 °C overnight. After washing with PBS, the cells were stained with Alexa Fluor 555 goat anti-rabbit IgG antibody (Thermo Fisher Scientific) for 1 h at 25 °C. After washing with PBS, the cells were further stained with Hoechst 33342 (Dojindo, Kumamoto, Japan) for 15 min, and the neurite length was measured using an IN Cell Analyzer 2200 (Cytiva, Tokyo, Japan), as previously described [9].

### 4.7. Measurement of Mitochondrial Activity

Cells were stained with 250 nM MitoTracker Red CMXRos (Thermo Fisher Scientific) at 37 °C for 30 min and subsequently with 200 nM MitoTracker Green FM (Thermo Fischer Scientific) at 37 °C for 30 min. Finally, the cells were stained with Hoechst 33342 at 37 °C for 30 min. Stained cells were analyzed using an IN Cell Analyzer 2200 to quantitatively determine the number, area, and activity of mitochondria using IN Cell Investigator high-content image analysis software ver. 6.0 (Cytiva, Tokyo, Japan) [18].

### 4.8. miRNA Microarray Analysis

The expression profiles of miRNAs in the exosomes were evaluated using microarray analysis with a 3D-Gene Mouse miRNA Oligo chip (Toray, Kamakura, Kanagawa, Japan). MiRNA preparation and subsequent operations were sourced from Kamakura Techno-Sciences, Inc. (Kamakura, Kanagawa, Japan). After global normalization of the miRNA expression levels, the ratio of each miRNA was calculated for comparison between the control and experimental samples. The criteria for regulated genes were then established, including a ≥2.0-fold ratio for upregulation [38]. The miRNA target genes were predicted using TargetScan (https://www.targetscan.org/vert_80/; accessed 3 March 2024). Tools and data provided by the Database for Annotation, Visualization, and Integrated Discovery (DAVID, https://david.ncifcrf.gov, accessed on 20 February 2024) were used to identify significantly enriched pathways [9,18,39,40].

### 4.9. Fluorescence Immunocytochemistry

Brain samples were collected from the heads of mice after the NORT was completed and fixed in 10% formalin buffer. Paraffin-embedded brain sections were prepared as described previously [41]. For immunohistochemical analysis, tissue sections were deparaffinized, rehydrated, and soaked in 1× HistoVT One (Nacalai Tesque, Kyoto, Japan). The sections were then heated at 90 °C for 20 min to activate the antigens. After washing with 0.1% Tween 20/TBS, the tissues were blocked with Blocking One Histo (Nacalai Tesque) for 1 h at 25 °C. Brain sections were incubated with primary antibodies for anti-Iba1 (#17197, Cell Signaling Technology, Danvers, MA, USA), anti-GFAP (#80788, Cell Signaling Technology), anti-p21 (#2974, Cell Signaling Technology), and anti-β Galactosidase (15518-1-AP, Proteintech, Rosemont, IL, USA), and subsequently with secondary antibodies (Alexa Fluor 555 anti-rabbit IgG, Thermo Fischer Scientific) [42]. After staining with Vectashield mounting medium (Vector Laboratories, Burlingame, CA, USA), tissue samples were observed under a fluorescence microscope (EVOS M5000 Cell Imaging System, Thermo Fischer Scientific) under identical exposure settings across all group [18]. For each animal, comparable rostrocaudal hippocampal sections were selected based on anatomical landmarks. Quantitative analyses were performed using ImageJ software verion 1.54 (https://imagej.nif.gov/ij, accessed on 20 February 2026). Regions of interest (ROIs) were defined within the hippocampal DG, CA1 and CA3 subregions. A consistent threshold value was used to identify positive signals. For Iba1 staining, positive cells were counted automatically and expressed as the number of positive cells per mm^2^. GFAP, p21 and SA-β-Gal immunoreactivity were quantified as the percentage of positively stained area relative to the total ROI area. All image acquisition and quantification procedures were performed in a blinded manner.

### 4.10. Quantitative Reverse Transcriptase-Polymerase Chain Reaction (RT-qPCR)

RNA was prepared from cells using a High Pure RNA Isolation kit (Roche Diagnostics GmbH, Mannheim, Germany) as described previously [43]. RT-qPCR was performed using the GoTaq 1-Step RT-PCR System (Promega, Madison, WI, USA) and Thermal Cycler Dice Real-Time System TP-800 (Takara, Kusatsu, Shiga, Japan). Samples were analyzed in triplicate. The PCR primer sequences used were as follows: mouse β-actin forward primer 5′-GGCCAGGTCATCACTATTG-3′ and reverse primer 5′-GAGGTCTTTACGGATGTCAAC-3′; mouse SIRT1 forward primer 5′-GCAGACGTGGTAATGTCCAAACAG-3′ and reverse primer 5′-GCAGACGTGGTAATGTCCAAACAG-3′; mouse peroxisome proliferator-activated receptor gamma coactivator 1-α (PGC1-α) forward primer 5′-CCGTAAATCTGCGGGATGATG-3′ and reverse primer 5′-CAGTTTCGTTCGACCTGCGTAA-3′; mouse nicotinamide phosphoribosyltransferase (NAMPT) forward primer 5′-CTCTTCGCAAGAGACTGCTGG-3′ and reverse primer 5′-CAGCAATTCCCGCCACAGTATC-3′; mouse transcription factor A, mitochondrial (TFAM) forward primer 5′-CATTTATCTATCTGAAAGCTTCC-3′ and reverse primer 5′-CTCTTCCCAAGACTTCATTTC-3′; and mouse neurofilament medium chain (NEFM) forward primer 5′-AGACAGACATCTCCACGGCG-3′ and reverse primer 5′-TGAACCACTCTTCGGCCTGG-3′. β-actin was used as a housekeeping gene. Samples were normalized and analyzed using the ΔΔCt method [44].

### 4.11. RNA Sequencing

RNA was extracted from the hippocampus using TRIzol Reagent (Thermo Fisher Scientific) and purified using the SV Total RNA Isolation System (Promega). RNA samples were quantified using an ND-1000 spectrophotometer (NanoDrop Technologies, Wilmington, DE, USA) and their quality was confirmed using TapeStation (Agilent Technologies, Inc., Santa Clara, CA, USA). Sequencing libraries were prepared using the MGIEasy rRNA Depletion Kit and MGIE RNA Directional Library Prep Set (MGI Tech Co., Ltd., Shenzhen, China). The libraries were sequenced on a DNBSEQ-G400 FAST Sequencer (MGI Tech) by the Bioengineering Lab (Atsugi, Kanagawa, Japan). Differential gene expression and pathway analyses were performed using the integrated iDEP web application (ver. 2.01, http://bioinformatics.sdstate.edu/idep/, accessed on 1 February 2024) [45]. The criteria for the differentially expressed genes were then established: *p* ≤ 0.05 and ratio ≥1.5-fold (upregulated genes) or 0.66-fold (downregulated genes) [18].

### 4.12. Statistical Analysis

Given the sample size and data distribution, non-parametric statistical analyses were applied. Group differences were evaluated using the Kruskal–Wallis test followed by Dunn’s multiple comparisons test with Holm correction. All tests were two-tailed, and *p* < 0.05 was considered statistically significant. In vitro experiments were performed using *n* = 3 biological replicates (independent cultures/animals). Each biological sample was measured in technical triplicate, and the mean of technical replicates was used for statistical analysis. The results are shown as the mean ± standard error.

## 5. Conclusions

This study demonstrates that exosomes play a central functional role in mediating the anti-aging effects of dietary GABA on the brain. Exosomes derived from GABA-fed aged mice effectively restored cognitive performance, enhanced mitochondrial activity, and suppressed neuroinflammation and cellular senescence in the hippocampus. These effects were accompanied by specific changes in exosomal miRNA profiles associated with neuronal activation, longevity, and energy metabolism. Our findings highlight exosomes as key biological mediators linking dietary factors to brain function. Thus, exosome-mediated signaling represents a promising therapeutic target for preventing age-related cognitive decline.

## Data Availability

The data that support the findings of this study are available from the corresponding author, Y.K. (Yoshinori Katakura), upon reasonable request. Study results can also be found in QIR (https://catalog.lib.kyushu-u.ac.jp/opac_browse/papers/?lang=0, accessed on 18 November 2025).

## Supplementary Materials

The following supporting information can be downloaded at: https://www.mdpi.com/article/10.3390/ijms27062519/s1.

## Abbreviations

The following abbreviations are used in this manuscript:

GABA	gamma-aminobutyric acid
miRNA	microRNA
NORT	novel object recognition test
DI	discrimination index
NAMPT	nicotinamide phosphoribosyltransferase
NEFM	neurofilament medium chain
RT-qPCR	quantitative reverse transcriptase–polymerase chain reaction
PGC1-α	peroxisome proliferator-activated receptor gamma coactivator 1-α
