# Supplementary material for: GABA-Induced Exosomes Improve Memory Impairment in Aged Mice
Source: Int J Mol Sci. 2026 Mar 10;27(6):2519. doi: 10.3390/ijms27062519 (PMC13026798; doi:10.3390/ijms27062519)
Supplement: Supplementary file 1 [file ijms-27-02519-s001.zip › ijms-4093829-supplementary.pdf]

Supplementary Figure S1

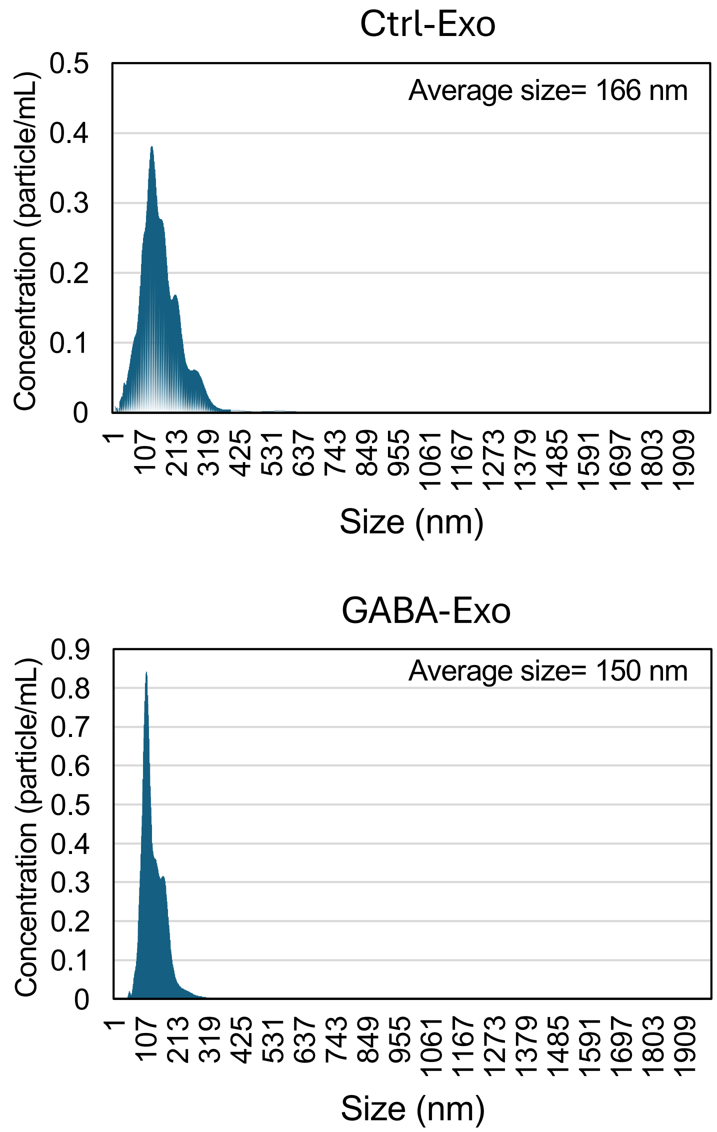

Figure S1. Size distribution of exosomes. Size distributions of plasma exosomes derived from Aged-Ctrl mice (a) and those from Aged-GABA mice (b) were analyzed by NanoSight (FUJIFILM). Particle size distribution was determined by nanoparticle tracking analysis (NTA), showing a typical exosomal size range.

Supplementary Figure S2

a

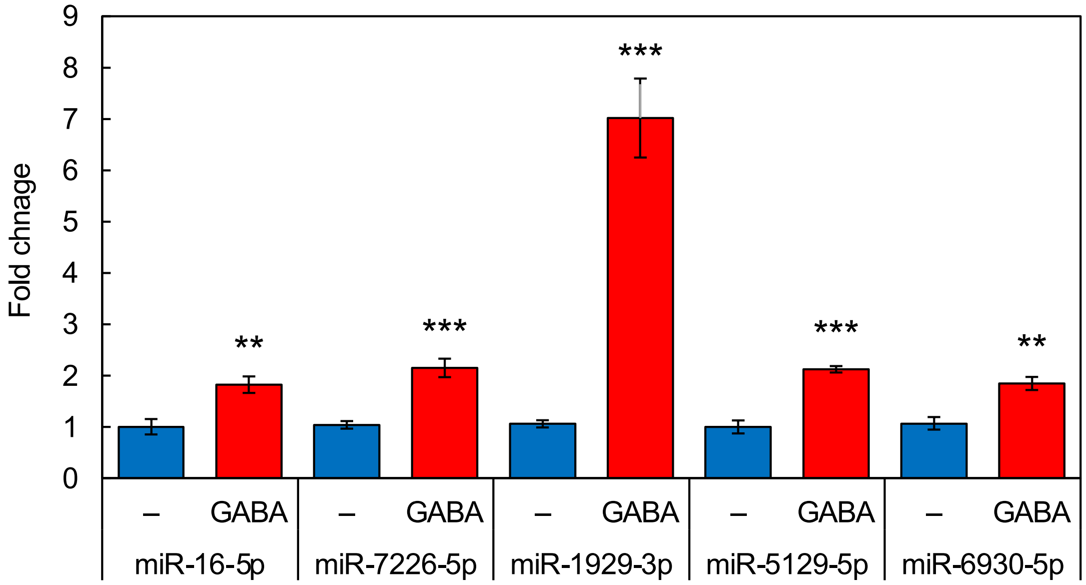

b

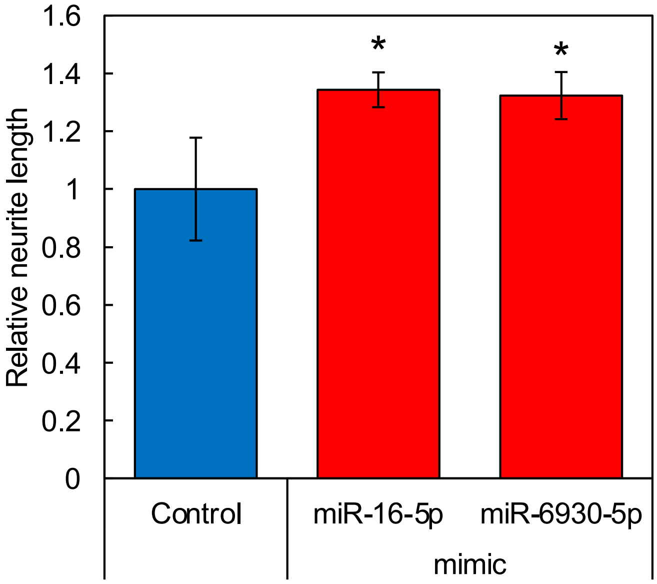

Figure S2. Expression and functional analysis of miRNA. a, Expression changes of selected miRNAs in exosomes derived from Aged-GABA mice (GABA) with those in exosomes derived from Aged-Ctrl mice (–). Total RNA in the exosomes was isolated using the miRNeasy Tissue/Cells Advanced Micro Kit (Qiagen). RT-qPCR was performed using the Mir-miRNA First-Strand Synthesis Kit (Takara) and TB Green Advantage qPCR Premix (Takara) according to the manufacturer’s protocol. b, Neurite length in SH-SY5Y cells transduced with miR-16-5p mimic or miR-6930-5p mimic was determined by using the IN Cell Analyzer 2200.

Supplementary Figure S3

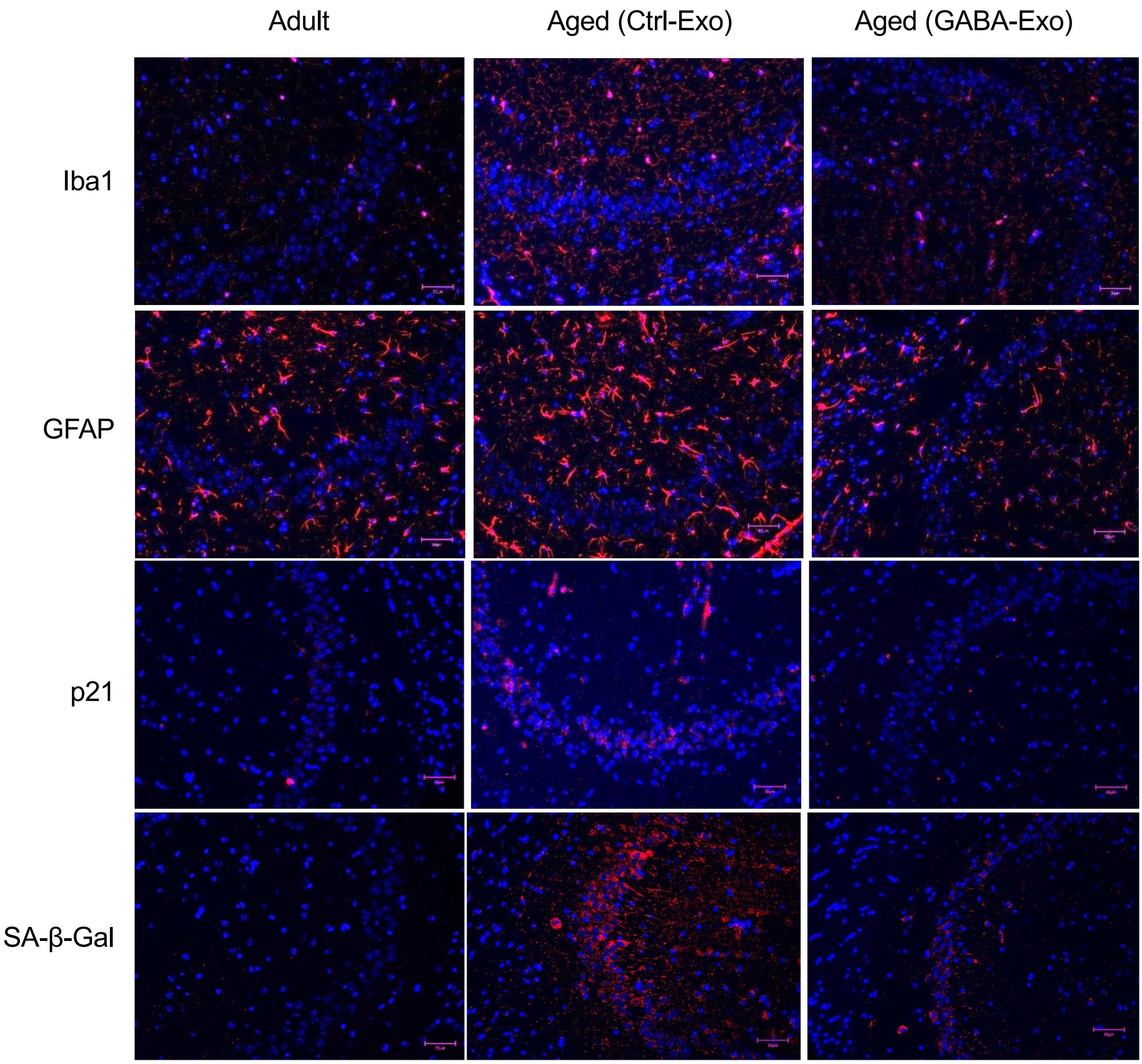

Figure S3. Effects of exosomes derived from GABA-ingested mice on hippocampus in aged mice. a, Brain sections (CA3) were incubated with primary antibody for anti-Iba1, anti-GFAP, anti-p21 and anti-β-galactosidase, and stained with Alexa Fluor 555.

Supplementary Figure S4

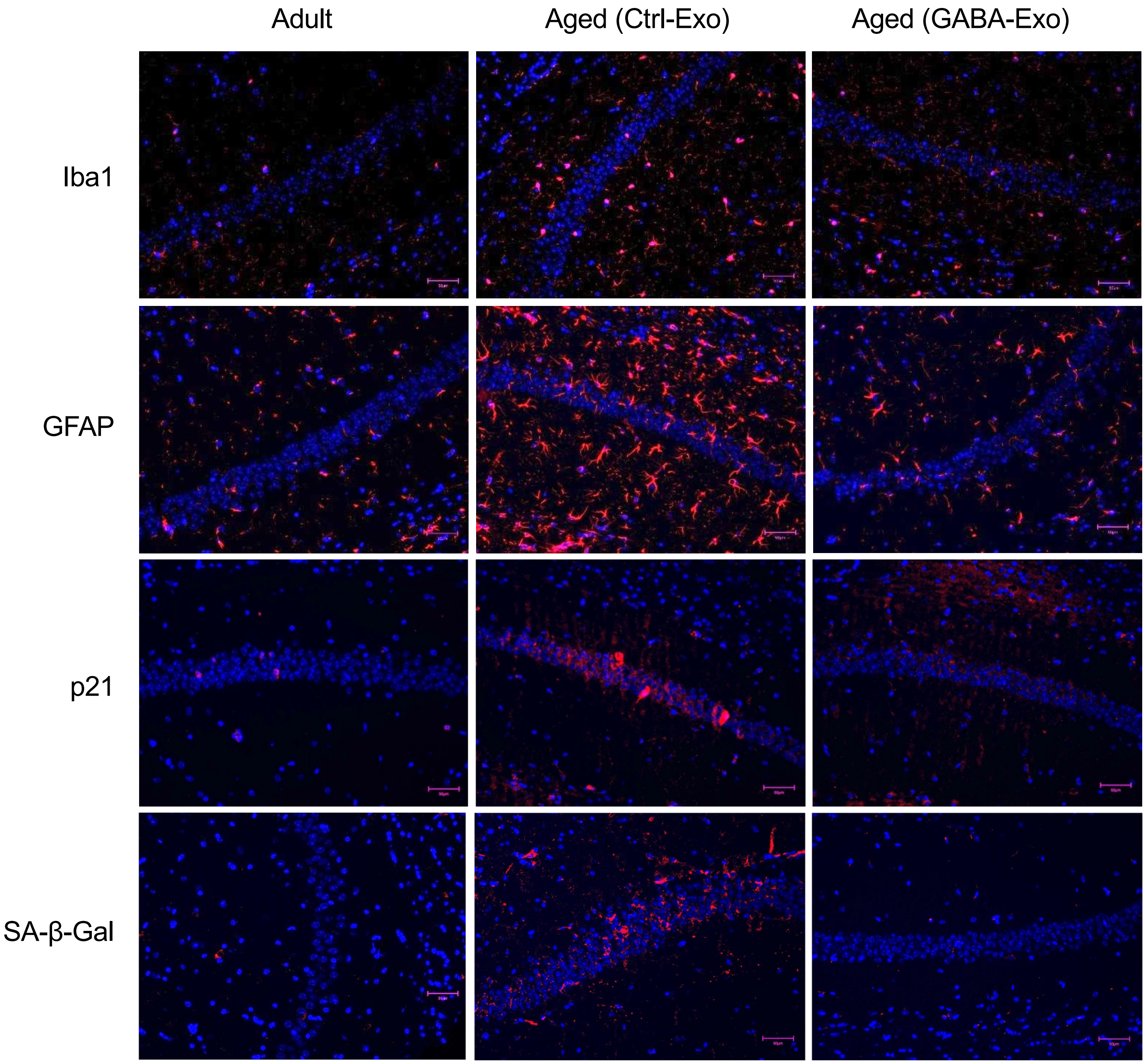

Figure S4. Effects of exosomes derived from GABA-ingested mice on hippocampus in aged mice. a, Brain sections (CA1) were incubated with primary antibody for anti-Iba1, anti-GFAP, anti-p21 and anti-β-galactosidase, and stained with Alexa Fluor 555.

Supplementary Figure S5

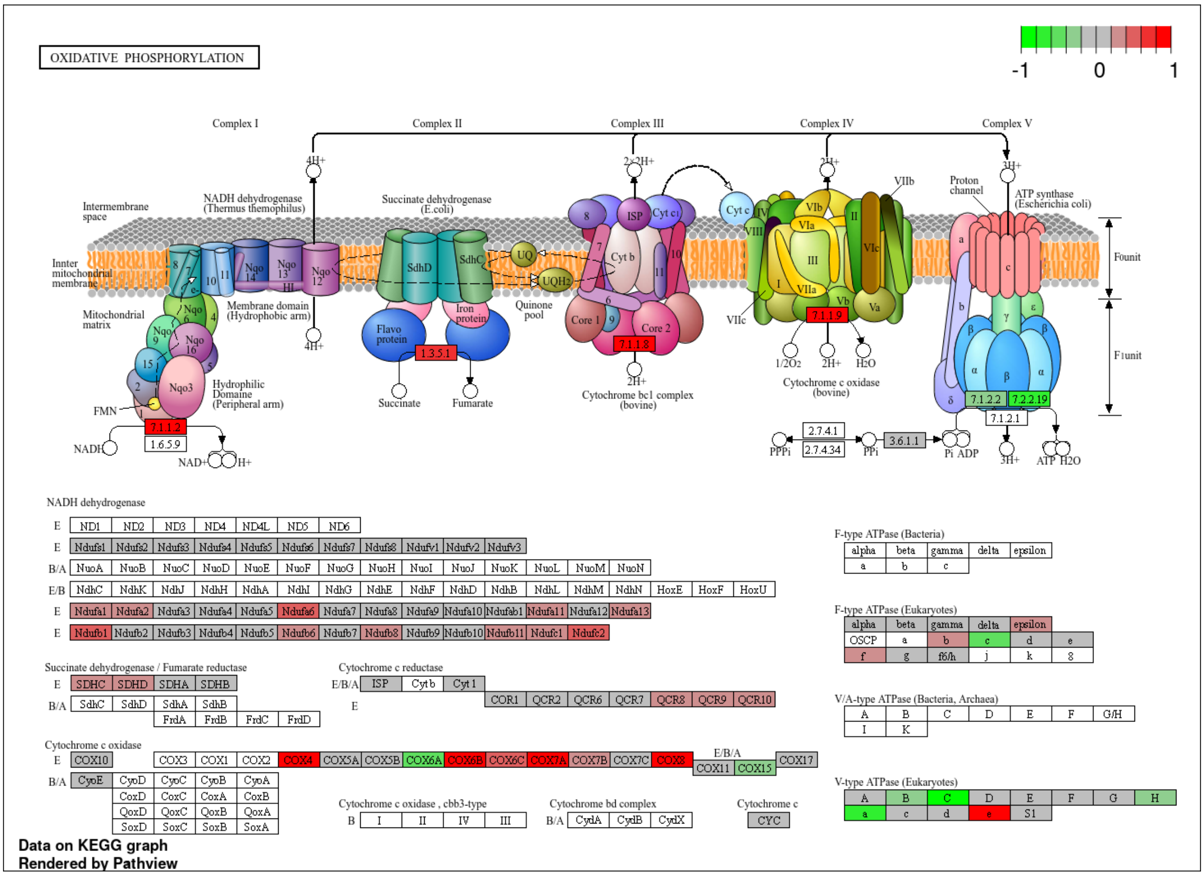

Figure S5. Comprehensive analysis of gene expression in the hippocampus. RNA-seq analysis revealed a change in gene expression in the hippocampus of Aged (GABA-Exo) compared with that of Aged (Ctrl-Exo). Many of the differentially expressed genes were involved in the oxidative phosphorylation pathway.

Supplementary Figure S6

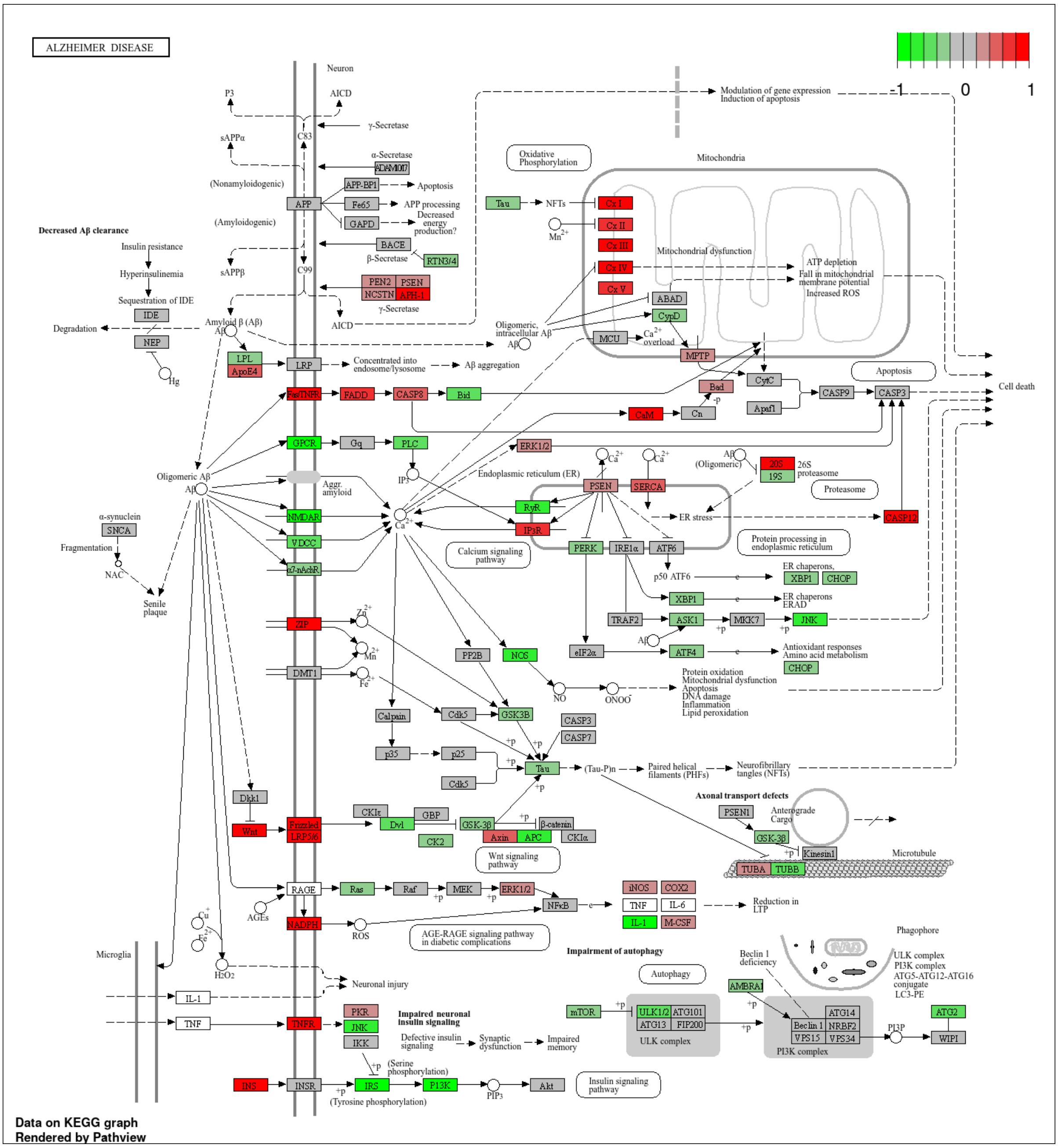

Figure S6. Comprehensive analysis of gene expression in the hippocampus. RNA-seq analysis revealed a change in gene expression in the hippocampus of Aged (GABA-Exo) compared with that of Aged (Ctrl-Exo). Many of the differentially expressed genes were involved in the Alzheimer's disease pathway.

Supplementary Figure S7

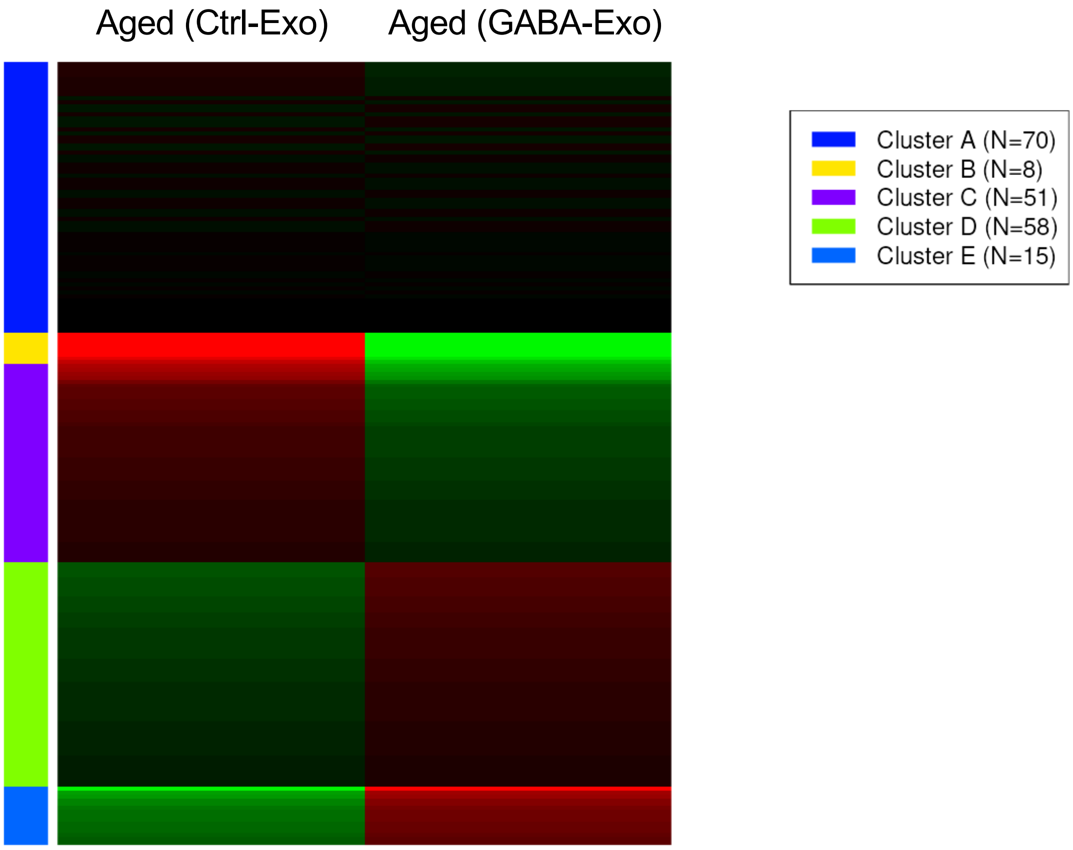

Cluster	p-value	Genes	Pathway
A	0.008	10	Modification-dependent macromolecule catabolic proces
E	0.002	2	Positive regulation of glycogen biosynthetic process
E	0.003	2	Regulation of calcineurin-mediated signaling
E	0.003	2	Glycogen biosynthetic process
E	0.003	2	Glucan biosynthetic process
E	0.003	2	Regulation of glucan biosynthetic process
E	0.003	2	Regulation of polysaccharide metabolic process
E	0.003	2	Calcineurin-NFAT signaling cascade
E	0.003	2	Regulation of calcineurin-NFAT signaling cascade
E	0.003	2	Calcineurin-mediated signaling
E	0.003	2	Positive regulation of glucose metabolic process
E	0.003	2	Inositol phosphate-mediated signaling
E	0.004	2	Glycogen metabolic process
E	0.004	2	Cellular glucan metabolic process
E	0.004	2	Cellular polysaccharide biosynthetic process
E	0.004	2	Glucan metabolic process

Figure S7. Comprehensive analysis of gene expression in the hippocampus. RNA-seq analysis revealed a change in gene expression in the hippocampus of Aged (GABA-Exo) compared with that of Aged (Ctrl-Exo). Differential gene expression and pathway analyses were performed using the integrated iDEP web application (ver. 2.01, http://bioinformatics.sdstate.edu/idep/, accessed on February 1, 2024) .
